# P-803. Utility of Respiratory Culture Gram Stain for De-Escalation of Empiric Antibiotics Against Methicillin-Resistant Staphylococcus aureus and Pseudomonas aeruginosa

**DOI:** 10.1093/ofid/ofaf695.1012

**Published:** 2026-01-11

**Authors:** Curtis Sera, Niki Arab, Brian Kim, Hera Maryam, Arthur Jeng

**Affiliations:** Olive View-UCLA Medical Center, Sylmar, California; Olive View- UCLA Medical Center, Los Angeles, California; Olive View-UCLA Medical Center, Sylmar, California; UCLA, Los Angeles, California; Olive View UCLA Medical Center/UCLA School of Medicine, Sylmar, California

## Abstract

**Background:**

Gram stain (GS) and BioFire® FilmArray® Pneumonia Panel (PN) are rapid methods of detecting bacterial pneumonia pathogens. Studies suggest that GS may aid early antibiotic de-escalation for infections caused by *Pseudomonas aeruginosa* (PsA) or methicillin-resistant *Staphylococcus aureus* (MRSA). However, PN is a highly sensitive and specific diagnostic tool for rapidly identifying numerous organisms validated for use on all types of respiratory specimens. The purpose of this study is to evaluate the sensitivity of GS vs. PN and GS vs. bronchoalveolar lavage culture (BAL cx) to determine the utility of GS for antibiotic de-escalation.

**Figure 1:**
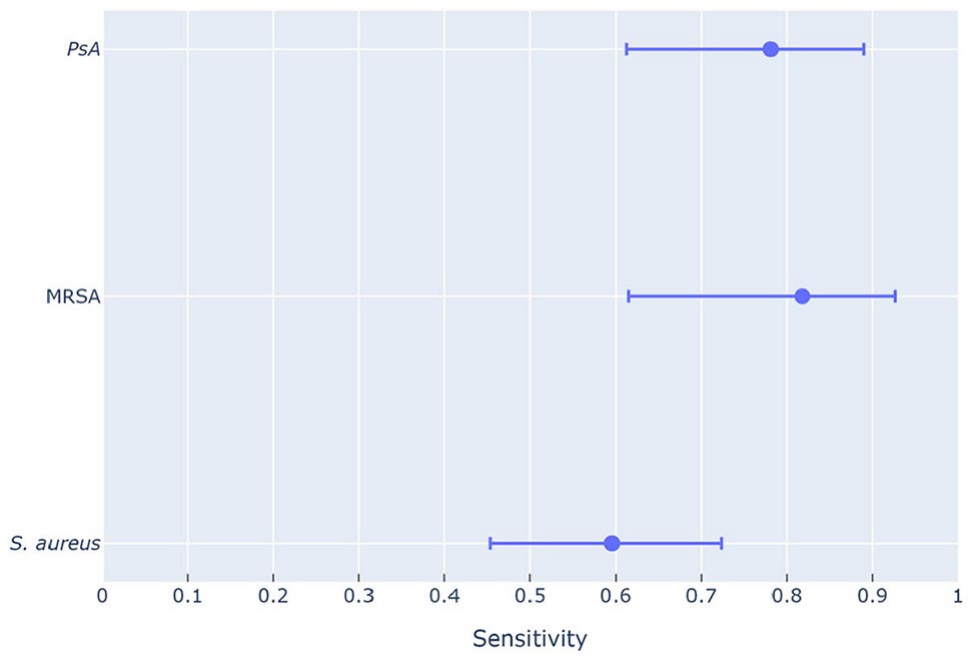
Gram stain vs BioFire® FilmArray® Pneumonia Panel sensitivity Compared to BioFire® FilmArray® Pneumonia Panel, Gram stain had sensitivities (95% confidence interval) of 78%, 82%, and 60% for Pseudomonas aeruginosa (PsA), methicillin-resistant Staphylococcus aureus (MRSA), and Staphylococcus aureus (S. aureus) respectively.

**Figure 2:**
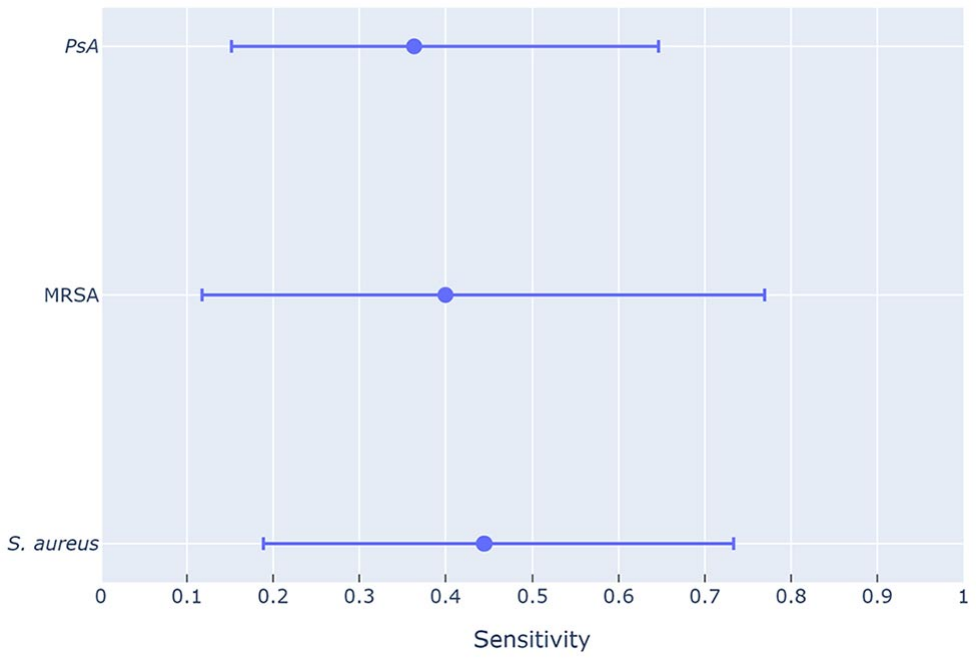
Gram stain vs bronchoalveolar lavage culture sensitivity Compared to formal bronchoalveolar lavage culture specimens, Gram stain had sensitivities of 36%, 40%, and 44% for Pseudomonas aeruginosa (PsA), methicillin-resistant Staphylococcus aureus (MRSA), and Staphylococcus aureus (S. aureus) respectively.

**Methods:**

This is a single center retrospective study from 1/2021-1/2024 on adults ≥18 years of age admitted for pneumonia. Respiratory sample GS sensitivity for detecting MRSA and PsA was measured using PN or BAL cx on the same specimen as gold standards. PN results were pooled for all respiratory specimen types (e.g. expectorated sputum, tracheal aspirate, BAL). Statistics were performed using Wilson 95% confidence intervals.

**Results:**

149 PN results and 59 BAL cx samples were compared to their corresponding GS. Compared to PN, the sensitivity of GS was 82% (95% confidence interval [CI]: 61% to 93%) for MRSA and 78% (95% CI: 61% to 89%) for PsA [Figure 1]. When compared to BAL cx, GS sensitivity was 40% (95% CI: 12% to 77%) for MRSA and 36% (95% CI: 15% to 65%) for PsA [Figure 2].

**Conclusion:**

GS appeared more sensitive compared to PN than to BAL cx as respective gold standards, but direct comparison cannot be made due to mixed specimen types in the PN group. GS on BAL samples had poor sensitivity compared to BAL cx for both MRSA and PsA detection, missing approximately 60% of both MRSA and PsA. Although GS sensitivity vs. PN appeared fair, GS would still miss approximately 20% of true pathogens. These findings raise clinical concern if GS alone was relied upon for de-escalating antibiotics, especially in critically ill patients.

**Disclosures:**

All Authors: No reported disclosures

